# Evolution of Plant Nucleotide-Sugar Interconversion Enzymes

**DOI:** 10.1371/journal.pone.0027995

**Published:** 2011-11-18

**Authors:** Yanbin Yin, Jinling Huang, Xiaogang Gu, Maor Bar-Peled, Ying Xu

**Affiliations:** 1 Computational System Biology Lab, Department of Biochemistry and Molecular Biology, and Institute of Bioinformatics, University of Georgia, Athens, Georgia, United States of America; 2 Complex Carbohydrate Research Center, University of Georgia, Athens, Georgia, United States of America; 3 Department of Biology, East Carolina University, Greenville, North Carolina, United States of America; 4 BioEnergy Science Center, Oak Ridge, Tennessee, United States of America; 5 College of Computer Science and Technology, Jilin University, Changchun, Jilin, China; American University in Cairo, Egypt

## Abstract

Nucleotide-diphospho-sugars (NDP-sugars) are the building blocks of diverse polysaccharides and glycoconjugates in all organisms. In plants, 11 families of NDP-sugar interconversion enzymes (NSEs) have been identified, each of which interconverts one NDP-sugar to another. While the functions of these enzyme families have been characterized in various plants, very little is known about their evolution and origin. Our phylogenetic analyses indicate that all the 11 plant NSE families are distantly related and most of them originated from different progenitor genes, which have already diverged in ancient prokaryotes. For instance, all NSE families are found in the lower land plant mosses and most of them are also found in aquatic algae, implicating that they have already evolved to be capable of synthesizing all the 11 different NDP-sugars. Particularly interesting is that the evolution of RHM (UDP-L-rhamnose synthase) manifests the fusion of genes of three enzymatic activities in early eukaryotes in a rather intriguing manner. The plant NRS/ER (nucleotide-rhamnose synthase/epimerase-reductase), on the other hand, evolved much later from the ancient plant RHMs through losing the N-terminal domain. Based on these findings, an evolutionary model is proposed to explain the origin and evolution of different NSE families. For instance, the UGlcAE (UDP-D-glucuronic acid 4-epimerase) family is suggested to have evolved from some chlamydial bacteria. Our data also show considerably higher sequence diversity among NSE-like genes in modern prokaryotes, consistent with the higher sugar diversity found in prokaryotes. All the NSE families are widely found in plants and algae containing carbohydrate-rich cell walls, while sporadically found in animals, fungi and other eukaryotes, which do not have or have cell walls with distinct compositions. Results of this study were shown to be highly useful for identifying unknown genes for further experimental characterization to determine their functions in the synthesis of diverse glycosylated molecules.

## Introduction

**Figure 1:**
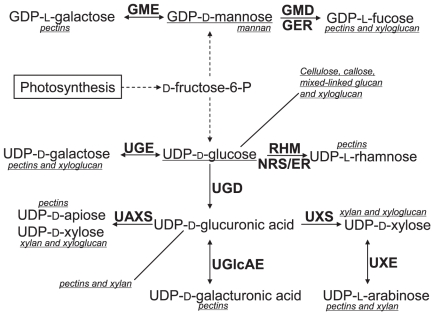
A partial list of plant NDP-sugars and interconversion enzymes. Eleven NDP-sugars and enzyme families involved in building plant cell wall polysaccharides are indicated. Polysaccharides in which NDP-sugars may be incorporated are indicated beside the respective NDP-sugar, underlined and italicized. Reactions are shown as arrows, and enzymes are indicated in bold beside the arrows. Abbreviations: UAXS (UDP-D-apiose/UDP-D-xylose synthase, also known as AXS), UGlcAE (UDP-D-glucuronic acid 4-epimerase, also know as GAE), GER (GDP-4-keto-6-deoxy-D-mannose-3,5-epimerase-4-reductase), GMD (GDP-D-mannose-4,6-dehydratase), GME (GDP-D-mannose 3,5-epimerase), RHM (UDP-L-rhamnose synthase), NRS/ER (nucleotide-rhamnose synthase/epimerase-reductase, also known as UER), UGD (UDP-D-glucose dehydrogenase), UGE (UDP-D-glucose 4-epimerase), UXE (UDP-D-xylose 4-epimerase) and UXS (UDP-D-xylose synthase, including AUD [membrane-anchored UXS] and SUD [soluble UXS]).

Nucleotide-diphospho-sugars (NDP-sugars) [1] are activated monosaccharide units that can be directly used by glycosyltransferases for synthesis of various glycoconjugates and polysaccharides. In plants there are at least 30 different NDP-sugars [1], [2], many of which have been implicated for their roles in the synthesis of different cell wall polysaccharides [2], [3], the major components of plant biomass, as depicted in Figure 1. Plant cell walls have recently received significant public attention due to their potential use as feedstocks for the next generation biofuel production [4] as part of the “green” effort to produce alternative energy.

NDP-sugars are mainly synthesized from fructose-6-phosphate, a product of photosynthesis. Among various NDP-sugars involved in the synthesis of plant polysaccharides, UDP-glucose and GDP-mannose can be produced from fructose-6-P, while other NDP-sugars are converted from either UDP-glucose or GDP-mannose through different epimerization, decarboxylation or dehydrogenation reactions [1], [2], [5], [6], [7]. Enzymes involved in these reactions are termed NDP-sugar interconversion enzymes (NSEs), as shown in Figure 1. In addition to the interconversion pathway, NDP-sugars can also be directly generated from free sugars through alternative pathways [5], [8], such as the salvage pathway [9], [10] to recycle free sugars released from cell wall degradation [2], or via other competing pathways [11], [12], which will not be described in this study. Recently, RGPs (Reversibly Glycosylated Proteins) were shown to interconvert UDP-L-arabinopyranose (UDP-Arap) and UDP-L-arabinofuranose (UDP-Araf) [13], implicating that more NSEs might be discovered in the near future.

All the NSEs shown in Figure 1 have been experimentally studied in either Arabidopsis or other plants [14], [15], [16], [17], [18], [19], [20], [21], [22], [23], [24], [25], [26], [27]. However, little is known about how the different plant enzyme families evolved and if they are evolutionarily related, considering that they catalyze a series of biochemical reactions that convert one type of very similar NDP-sugar to another. If they are related, there remain fundamental evolutionary questions to be answered: when did they diverge and where did they originate from?

We have computationally identified NSE homologs from different sources including four fully sequenced plant and algal genomes (*Chlamydomonas reinhardtii* [28] [unicellular *Chlorophyta* green alga belonging to *Viridiplantae* (green plant)], *Physcomitrella patens ssp patens* [29] [moss], *Oryza sativa* [30], [31] [monocot] and *Arabidopsis thaliana* [dicot] [32]), NCBI-nr database and assembled EST unique transcripts of PlantGDB [33]. The homology search revealed much higher sequence diversity for NSE homologs in prokaryotes than in plants, consistent with the fact that more monosaccharides are found in prokaryotes than other organisms [34]. Orthologs of all NSE families are explicitly found in eukaryotes with carbohydrate-rich cell walls such as plants and various algae. Our phylogenetic analyses indicate that plant NSEs belong to a very large and ancient gene superfamily. Ancestors of this superfamily have evolved and diverged in ancient prokaryotes to give rise to numerous gene families including NSEs before eukaryotes appeared; some of these gene families were then transferred into ancient eukaryotic cells through either vertical inheritance from direct ancestors or horizontal gene transfers from other ancient prokaryotes including endosymbiotic gene transfers.

## Results

Thirty-six Arabidopsis genes were predicted to encode NSEs forming 11 enzyme families [6] (see Fig. 1 for details). These families fall into six classes according to their biochemical activities: 4-epimerases (UGlcAE [GAE], UGE and UXE; see Fig. 1 for the full names), 3,5-epimerases (GME), 3,5-epimerases-4-reductases (GER, RHM-C-terminal region and NRS/ER [UER]), 4,6-dehydratases (GMD and RHM-N-terminal region), decarboxylases (UAXS [AXS] and UXS) and 6-dehydrogenases (UGD). Thirty-two of the 36 Arabidopsis proteins contain the Pfam *Epimerase* domain (Pfam short description: *NAD dependent epimerase/dehydratase family*, accession number: PF01370, length: 286 aa) while the four UGD proteins do not. Unlike the other NSEs that contain only one domain, RHM proteins comprise of two distinct catalytic domains fused into one large polypeptide: the N-terminal domain with 4,6-dehydratase activity and the C-terminal domain with 3,5-epimerase-4-reductase activity [25], [27].

### Plant NSE families have diverged anciently

**Figure 2:**
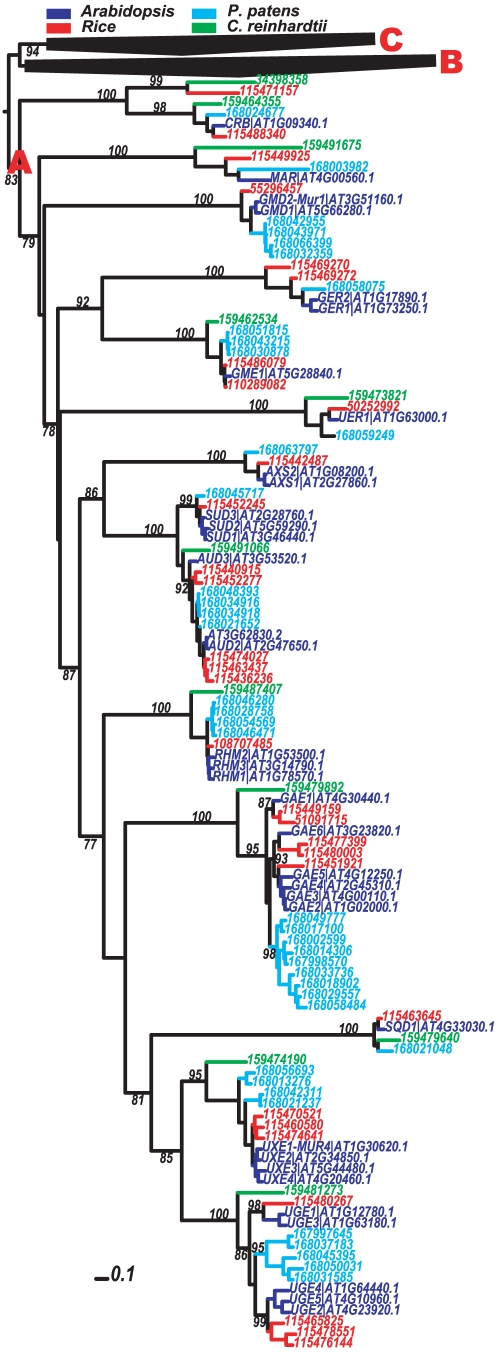
Phylogeny of 257 plant Epimerase domains. The phylogeny is built using PhyML v3.0 and displayed using the Interactive Tree of Life (iTOL) web server (Letunic and Bork, 2007). Bootstrap values beside the nodes indicate the confidence levels with regard to the clustering of relevant proteins into one group. Selected supporting values >70% are shown. SQD1 is UDP-sulfoquinovose synthase. MAR is short for methionine adenosyltransferase regulatory protein, whose exact enzymatic function is not determined yet. CRB is short for chloroplast RNA binding. For other names, see Figure 1 for abbreviations. Note that UXS includes SUD and AUD, UGlcAE is also known as GAE, UAXS is also known as AXS and NRS/ER is also known as UER. Only sub-clades of major clade A are shown and sequence names are indicated using GenBank gi numbers or UniGene IDs. The other two clades are collapsed as black triangles. The scale bar corresponds to 0.1 changes per amino acid position. The complete version of this phylogeny is given in Figure S1.

Homology searches (E-value <0.01) found 257 *Epimerase* domain-bearing proteins from four sequenced plant and algal genomes, 22,547 from the NCBI-nr database and 488 from the assembled plant EST database PlantGDB. As shown in Figure 2 and Figure S1, the 257 plant *Epimerase* domains form three major clades in the phylogeny. Clade A contains 13 sub-clades consisting of 117 proteins among which 35 are from Arabidopsis. Thirty-two out of the 35 proteins are from ten NSE families: UXS, UAXS, UGlcAE, UGE, UXE, RHM-N-terminal, GME, GER, GMD and NRS/ER. The remaining three proteins are the UDP-sulfoquinovose synthase (SQD1 [35], AT4G33030), the chloroplast RNA binding protein (CRB, AT1G09340) and an uncharacterized protein (AT4G00560) annotated as “methionine adenosyltransferase regulatory beta subunit-related” by TAIR (The Arabidopsis Information Resource) [36], which is termed as the MAR family (sub-clade) in our analysis. Among them the SQD1 sub-clade is clustered with the UXE and UGE sub-clades, while the MAR and CRB seem to be just distantly related to the NSE families.

It is clear from the phylogeny (Fig. 2 and Figure S1) that all the 13 sub-clades in clade A have representative proteins from Arabidopsis, rice and *P. patens,* and ten of the 13 sub-clades also have representative proteins from the green algal *C. reinhardtii*. Further investigation of the EST homologs confirms that all the 13 sub-clades are present in gymnosperms as well. Separate searches found that NSEs except for GMD, GER, UAXS and UXE are also found in unicellular red algal *Cyanidioschyzon merolae* genome [37] and all except for UXE are also found in multi-cellular brown algal *Ectocarpus siliculosus* genome [38]. Hence plant NSE families must have diverged from each other at latest before the appearance of unicellular algae.

To further investigate the divergence point of the ancestors of the 13 sub-clades containing the ten plant NSE families, hidden Markov models (HMMs) were generated (see Methods for details) to represent the 13 sub-clades of clade A and the other two major clades B (45 sequences) and C (95 sequences), respectively. The 15 plant HMMs were then used to search against the 22,547 NCBI-nr *Epimerase* domain sequences in order to classify them into the 13 groups, each containing sequences more similar to the corresponding HMM than to the other HMMs. Table 1 shows that each HMM retrieves NCBI-nr proteins from various organisms including plants, animals, fungi, bacteria and archaea (see Tables S3, S4, S5, S6, S7, S8, S9, S10, S11, S12, S13, S14, S15 and S16 for the list of included proteins). This means that the 11 plant NSE families are closer to their non-plant homologs than to each other, hence suggesting that these families have split from each other very anciently before the earliest eukaryotes emerged. In addition, the presence or absence of homologs of the 11 NSE families shown in Table 1 also reflects the presence or absence of some particular sugars in certain organisms. For example, mammals do not have any close homolog of UAXS, consistent with the fact mammals do not contain apiose [34].

**Table 1:** Numbers of close NCBI-nr homologs of the respective plant NSE families.

Organisms	RHM-N	NRS/ER	UXS	UAXS	UGE	UXE	SQD1	UGlcAE	GER	GME	GMD	MAR	CRB
***Viridiplantae***	36	49	111	26	86	51	23	86	28	53	38	21	24
***Fungi***	33	9	12	1	105	12	0	1	3	2	3	36	1
***Metazoa***	38	4	60	0	92	37	0	10	84	1	81	42	2
***Other euk.***	21	8	17	0	35	1	3	13	20	5	22	6	8
***Archaea***	77	0	125	0	17	16	20	21	6	2	28	56	0
***Bacteria***	2989	4	1073	170	1930	902	126	923	573	43	766	1132	195

### Plant NSE families have different prokaryotic progenitors

**Figure 3:**
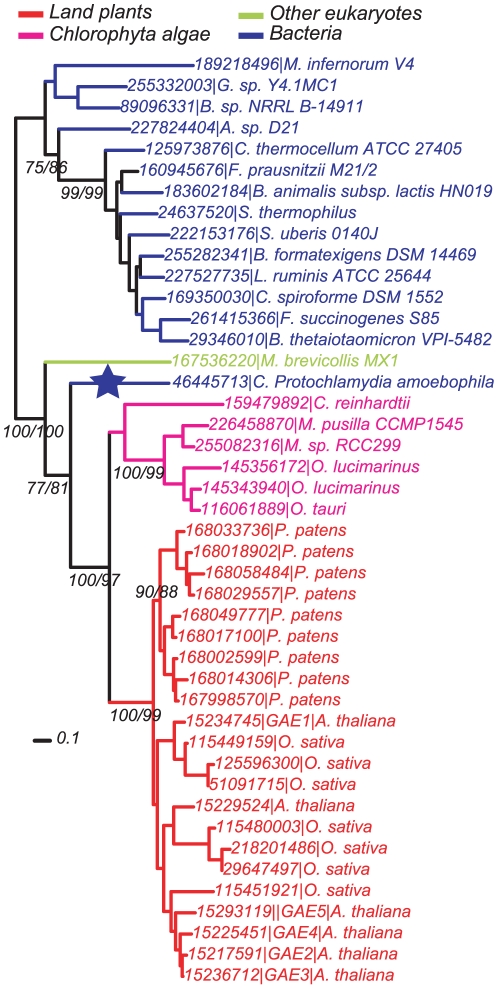
Phylogeny of 44 Epimerase domains closest to plant UGlcAE proteins. The 44 sequences are shown with GenBank gi numbers followed by species names. The phylogeny is built using both PhyML v3.0 and FastTree v2.1.1 and displayed using the Interactive Tree of Life (iTOL) web server. The topology by PhyML is shown and selected supporting values >70% from PhyML and FastTree analysis are indicated and split by ‘/’. Blue star indicates the closest bacterial homolog of plant UGlcAE proteins.

It has been well-documented that the earliest eukaryotic cell evolved from ancient prokaryotes [39], [40] and that most of the prokaryotic phyla are much more ancient than any eukaryotes [41], [42]. Hence we infer that if some eukaryotic genes are clustered together with prokaryotic genes of diverse organisms in a gene phylogeny, the later should in general be related to the origin of the former (except for a few very rare cases of recent gene transfers from bacteria to higher eukaryotes). Figure 3 shows that the plant UGlcAE family is clustered (supporting value  = 100%) with two GenBank proteins, one (gi#: 46445713) from a chlamydial species *Candidatus Protochlamydia amoebophila* UWE25 and the other from a unicellular eukaryotic species *Monosiga brevicollis* MX1 (gi#: 167536220) (also see Figure S2, red fonts). Many modern chlamydial bacteria are symbionts of various eukaryotic hosts [43], and the ancient chlamydial bacteria may have contributed a significant number of genes to the ancient plant cell [44], [45]. It is thus not surprising that they may have also contributed to the origin of the plant UGlcAE family. To validate this finding it would be very interesting to experimentally examine if this modern chlamydial protein (gi#: 46445713) also carries the UGlcAE activity.

Similarly, we also examined the phylogenies of the other plant NSE families including UGD, which are given in Figures S3, S4, S5, S6, S7, S8, S9, S10, S11, S12, S13, S14 and S15. Information of proteins included in these phylogenies is available in Tables S3, S4, S5, S6, S7, S8, S9, S10, S11, S12, S13, S14, S15 and S16. Again the plant NSE proteins in each of these phylogenies are more similar to the prokaryotic proteins in the same phylogeny than to the other plant families. The closest prokaryotic species to the plant NSEs are identified in each phylogeny and listed in Table S1, to be the putative prokaryotic progenitors of the respective plant NSE family.

### Phylogenies help pinpoint interesting proteins for experimental characterization

Phylogenies shown in Figures S2, S3, S4, S5, S6, S7, S8, S9, S10, S11, S12, S13, S14, S15 and data presented in Tables S3, S4, S5, S6, S7, S8, S9, S10, S11, S12, S13, S14, S15 and S16 are also very helpful in identifying uncharacterized proteins for further biochemical investigation. Functionally unknown proteins from non-plant organisms that are close to plant NSEs in the phylogenies may carry the similar biochemical activities. For instance, UXS enzymes have been characterized in fungi, plant and animal, but never in bacteria and archaea. We recently selected two bacterial proteins (gi#s: 262189116/16264188 and 262189118/16263977, red fonts in Figure S5) from *Sinorhizobium meliloti* and one protein (gi#: 88188828/88603366) from an archaeal species *Methanospirillum hungatei* (Bar-peled et al., unpublished data), close to plant UXS proteins in the phylogeny, and showed that they all carry the UXS activity [46]. We also characterized a bacterial protein (gi#: 293339156/152974263) from *Ralstonia solanacearum*, phylogenetically located between the plant UXSs and UAXSs, to be a bifunctional UDP-4-keto-pentose/UDP-xylose synthase [47].

Another example is from UGE and UXE-like proteins. In plants the UGE proteins form two separate sub-clades (Figure 2), one of which is promiscuous and possesses not only UGE but also UXE activities [48], and the other has a strict UGE activity. Interestingly, we found that one bacterial protein (gi#: 49182215/30265469, BAS5304, red fonts in Figure S13) close to plant UGEs in our phylogeny, was documented to have the similar promiscuity, which can not only convert UDP-Glc to UDP-Gal but also convert UDP-GlcNAc to UDP-GalNAc [49]. In addition, we selected a bacterial protein (gi#: 16264189, SmUXE) in the UGE-like gene list based on the phylogeny, and characterized it to have the UXE activity [46], providing the first evidence that bacteria also encode UXE activity.

These examples together demonstrate the power of phylogeny-based approach assisted with inspection of sequence alignments in helping experimental biologists to select gene targets and form testable hypothesis.

### Phylogenetic analyses of plant RHM and NRS/ER proteins

**Figure 4:**
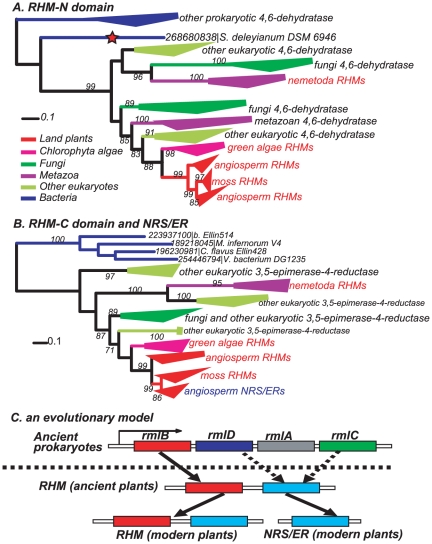
Phylogenies of 254 RHM N-terminal and 78 C-terminal domains. A) 254 sequences closest to plant RHM N-terminal domains. The red star indicates the closest bacterial homolog of eukaryotic 4,6-dehydratases. B) 78 sequences closest to plant RHM C-terminal domains and plant NRS/ER proteins; these sequences were obtained by searching a self-built plant NRS/ER HMM against the NCBI-nr database (E-value <1e-2). The phylogenies are built using FastTree v2.1.1 and displayed using the Interactive Tree of Life (iTOL) web server. Major clades are collapsed as triangles and selected supporting values >70% are shown. Un-collapsed sequences are indicated using GenBank gi numbers followed by species names. The complete phylogenies with un-collapsed clades are given in Figures S3 and S4. C) A proposed model for the evolutionary route of the bi-domain RHMs and the single-domain 3,5-epimerase-4-reductases (NRS/ERs) in plants. The prokaryotic gene cluster is an example from Salmonella enterica serovar Typhi CT18 (Parkhill et al., 2001). Note that in different bacteria the order of the four genes could vary and some of the genes could be missing or replaced by other genes.

As mentioned earlier, Arabidopsis RHM proteins have two domains [3], [25], [27]. The C-terminal domains do not match the Pfam *Epimerase* domain even using a rather relaxed cutoff, E-value <10. Nevertheless our self-built HMM based on plant NRS/ER *Epimerase* domains was able to detect the C-terminal domains of the RHM proteins because of the high sequence similarity between the NRS/ER proteins and the C-terminal domains of RHM. The phylogenies for the RHM N-terminal regions (Fig. 4A and Figure S3) and the C-terminal regions (Fig. 4B and Figure S4) include their homologs from the NCBI-nr database. Comparison between the two phylogenies indicates that the C-terminal domain has much fewer bacterial homologs than the N-terminal domain. Only four bacterial homologs were found for the C-terminal domain using the E-value cutoff <0.01, all from the *Verrucomicrobia* bacterial phylum. Interestingly *Verrucomicrobia* bacteria are closely related to *Chlamydiae* bacteria, which may have contributed many genes to ancient plants including the UGlcAE genes (see above). In contrast, hundreds of bacterial homologs were found for the N-terminal domain (collapsed as a blue triangle in Figure 4A). The possible reason for this discrepancy could be that the C-termini have diverged more substantially than the N-termini since the C-termini combined two different biochemical activities: 3,5-epimerase and 4-reductase.

Twenty-four proteins were found in both Figure 4A and 4B, indicating that they are bi-domain RHM proteins (red fonts in Fig. 4). Among them, 14 are from angiosperms, three from mosses, three from green algae and four from *Nematoda*. All the remaining sequences in the two phylogenies are single-domain proteins, carrying 4,6-dehydratase activity in Fig. 4A and 3,5-epimerase-4-reductase activity in Fig. 4B. In addition, a BLAST search using Arabidopsis RHM proteins as the query found ESTs of *Pinus taeda* and *Picea glauca* matched both domains, suggesting that the bi-domain RHMs are also present in gymnosperms. Hence the topology shown in Fig. 4B suggests that angiosperm NRS/ER proteins may be the result of an ancient duplication followed by losing the N-terminus of the duplicated RHM gene to become NRS/ER; otherwise the angiosperm NRS/ER proteins (blue fonts) should be clustered with other eukaryotic 3,5-epimerase-4-reductases in Fig. 4B. Although one green algal protein (gi #: 159473821) and one moss protein (gi#: 168059249) within the RHM clades are single-domain proteins (red fonts in Figure S4), it is very likely that these proteins either recently lost their N-termini or are mis-annotated.

Further comparison between the two phylogenies revealed that all other eukaryotic organisms in Fig. 4B (green and yellow green) are also found in Fig. 4A (also see Figure S3), indicating that they have both the 4,6-dehydratase and the 3,5-epimerase-4-reductase activities while as two separate genes, as opposed to what were found in plants and *Nematoda*. For example, the fungal pathogen *Botryotinia fuckeliana* B05.10 encode two separate proteins, one (gi#: 154311283, red fonts in Figure S4) having a high sequence identity and the similar enzymatic activity to that of the plant RHM N-terminal domains (Martinez, Smith, Bar-Peled , unpublished data) and the other (gi#: 154322248, red fonts in Figure S3) with highly similar sequence to plant RHM C-terminal regions and capable to form UDP-Rhamnose (Bar-peled et al., unpublished data).

## Discussion

### Evolution of plant RHM and NRS/ER proteins

The evolution of bi-domain RHM proteins and single-domain NRS/ER proteins presents a prominent example of gene fusions in early eukaryotes. The “RHM” equivalent activities for the formation of TDP-L-rhamnose are carried by three distinct genes: rmlB (4,6-dehydratase), rmlC (3,5-epimerase) and rmlD (4-reductase) genes in many prokaryotes (Figure 4C) [50], [51], [52]. It is thus tempting to speculate that prokaryotic rmlB gave rise to the N-terminal domains (4,6-dehydratase) of the eukaryotic RHM proteins, while rmlC and rmlD somehow evolved to become the C-terminal domains (3,5-epimerase-4-reductase).

Using the bacterial protein (red star in Figure 4A) closest to eukaryotic 4,6-dehydratases as a query, we searched against all fully sequenced prokaryotic genomes. For the top matched genes, we checked their synteny in their respective genomes, and found that in many bacterial genomes at least two of the three genes (4,6-dehydratase, 3,5-epimerase and 4-reductase) are clustered together within a region spanning seven genes (Tables S1 and S2). For example, in 70 out of 123 bacterial genomes, genes encoding 4,6-dehydratase and 3,5-epimerase are clustered together.

Based on the above observations, we proposed a model for the origin of plant RHMs and NRS/ERs (Fig. 4C). Specifically, the ancient eukaryotic cell acquired one DNA fragment (e.g. one bacterial operon) containing the three activities (carried by rmlB, C and D). In the donor prokaryotic organism, genes encoding the 3,5-epimerase and 4-reductase (rmlC and rmlD) activities may have already been “*integrated*” into one gene (3,5-epimerase-4-reductase). In the recipient eukaryotic cell, this gene was further fused with the neighboring 4,6-dehydratase gene into a larger gene encoding the ancient bi-functional RHM proteins, while the other genes in the fragment (e.g. rmlA: glucose-1-phosphate thymidylyltransferase) were lost or moved elsewhere in the chromosome.

It remains unknown how and when the earliest 3,5-epimerase-4-reductase gene emerged (dotted arrows in Fig. 4C). The fact that it has only four bacterial homologs across all the sequenced bacterial genomes suggests that the C-terminal domains of RHM have changed too much or all other prokaryotes bearing this gene are largely extinct. It is possible that the ancestral 3,5-epimerase-4-reductase has an earlier 3,5-epimerase ancestor or an earlier 4-reductase ancestor. This is supported by the fact that the GER proteins, which also possess the 3,5-epimerase-4-reductase activity, are phylogenetically closer to the 3,5-epimerase family GME (Fig. 2).

After the emergence of RHM genes in early eukaryotes, one of the two domains might have independently lost. For example, in early land plants (or more specifically in early angiosperms) the RHM gene was subject to one gene duplication; in one copy the N-terminal domain was lost, which eventually evolved to be the single-domain NRS/ER protein (Fig. 4B). Interestingly all other eukaryotes in Fig. 4B (except for *Nematoda*) encode both a 3,5-epimerase-4-reductase gene and a separate 4,6-dehydratase gene, possibly due to the loss of selection pressure that forced them to stay together. In contrast, all the remaining eukaryotes in Fig. 4A including some fungi and metazoa only encode 4,6-dehydratases, possibly because the C-terminal domains were lost. The simultaneous existence of bi-domain RHM proteins, single-domain 3,5-epimerase-4-reductases and single-domain 4,6-dehydratases in different eukaryotes implicates the very complex evolution of the RHM related proteins. Although the model presented in Fig. 4C is favored, which we confined to only plants, we do not rule out the alternative model, i.e. the ancient prokaryotic rmlB, rmlC and rmlD genes were independently introduced into early eukaryotes, and were independently fused into the bi-domain RHMs in ancient plants and *Nematoda*.

### An evolutionary model for plant NSEs

The NSE proteins that contain the Pfam *Epimerase* domain were previously classified to be of the short chain dehydrogenase/reductase (SDR) superfamily [53] that is also represented by a Pfam HMM, called the *adh_short* domain (Pfam short description: *short chain dehydrogenase*, accession number: PF00106, length: 181 aa). Both Pfam families (*Epimerase* and *adh_short*) belong to the *NADP_Rossmann* clan [7] (CL0063, Pfam description: *FAD/NAD(P)-binding Rossmann fold Superfamily*), which contains a total of 148 Pfam families (http://pfam.janelia.org/clan/NADP_Rossmann). A Pfam *clan* is a higher-level classification of protein sequences, covering multiple Pfam families sharing a common but distant evolutionary origin, which explains why many *Epimerase* domain-bearing proteins also match other Pfam domains such as the *adh_short* domain. In this sense the *NADP_Rossmann* clan groups all the plant NSE families together, as plant UGD proteins also belong to the Pfam *NADP_Rossmann* clan [7]. Proteins of this clan all bind with NAD/NADP/FAD as cofactors using the conserved Rossmann-fold domain in the N-termini, while their C-terminal domains bind diverse substrates such as sugars, alcohols, steroids, aromatic compounds and xenobiotics.

**Figure 5:**
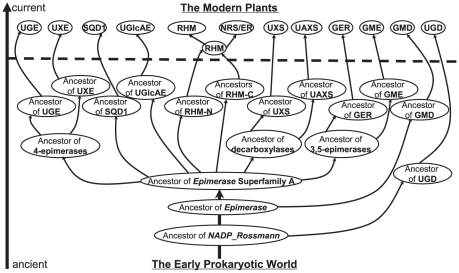
An evolutionary model for the origin of plant NSE families . The ancient prokaryotes include ancient bacteria and ancient Archaea. The thick horizontal dash line indicates the time when the earliest eukaryotes emerged. The arrows show the direction of evolution.

Hence, the following evolutionary model is proposed to explain the origin and evolution of all plant NSEs starting from the most ancient ancestor of the *NADP_Rossmann* clan in the early prokaryotic world (Figure 5). During evolution this ancestor gave rise to the ancient *Epimerase* domain, which should have already contained the conserved ATP/NAD/NADP binding motif GxxGxxG in their N-terminal region, commonly found in many families of the *NADP_Rossmann* clan. This earliest domain then diverged into three major superfamilies/clades A, B and C (Fig. 2 and Figure S1), among which A was the latest common ancestor of the ten NSE families.

The divergence of this superfamily A ancestor further led to the specialization of distinct enzyme activities: 4-epimerase, decarboxylase, 3,5-epimerase and 4,6-dehydratase, although the order of the divergence remains unknown. The ancestors of these activities further gave rise to the earliest prokaryotic NDP-sugar biosynthetic enzymes. Consistent with this, we found that plant enzymes of similar activity are often evolutionarily closer (Fig. 2), e.g. UGE and UXE (4-epimerases), GME and GER (3,5-epimerases), UAXS and UXS (decarboxylases).

It is interesting to note that bacteria produce considerably more diverse mono-saccharides than mammals and plants to build their capsules and cell walls [34]. This higher sugar diversity in modern prokaryotes is consistent with our finding that bacterial NSE homologs have considerably higher sequence diversity than eukaryotic NSEs (Table 1 and Tables S3, S4, S5, S6, S7, S8, S9, S10, S11, S12, S13, S14, S15 and S16). Many of the bacteria-specific clades have not yet been characterized, which might be responsible for synthesizing the unusual sugars not found in plants and animals. We demonstrated in this paper that the phylogenies generated in this study helped us to have characterized a number of unknown bacterial NSEs [46], [47] (and Bar-peled et al., in preparation). Moreover, we are in the process of building a sequence database for NSE homologs identified in this study, which could be valuable for biochemists to select interesting bacterial/fungal target genes for further functional characterization.

Recent reviews [54], [55], [56], [57] suggested that the primary endosymbiotic gene transfers (EGTs) [58] and other endosymbiotic events have played significant roles in the origin of numerous enzymes involved in plant cell wall synthesis, e.g. glycosyltransferases [59], [60], [61] and CMP-Kdo [62]. It is possible that different progenitor genes of plant NSEs were also introduced into plant cells through these ancient endosymbioses or through other horizontal gene transfers that happened in the early eukaryotes or plants [52], [63], [64]. It is generally believed that for unicellular organisms horizontal gene transfers between cells through phagocytosis, virus infection, intimate association or other processes were very frequent [52]. However, it remains a mystery as to which NSEs entered the ancient plant genome after EGTs and which NSEs were individually acquired from other bacteria. Interestingly most NSEs have a clear ortholog in *C. reinhardtii* of the *Chlorophyta* green algae, which contains charophycean where all land plants have evolved. It is thus tempting to speculate that the ancient “plant-like” cells have integrated all NSEs at latest before unicellular green algae (*Chlorophyta*) appeared. Hence ancient cells earlier than aquatic algae might have already been able to synthesize most the 11 cell wall related NDP-sugars, although modern algae, e.g. *C. reinhardtii* and *C. merolae*, may have lost some of the NSE genes.

Recently the genome of the multi-cellular brown alga *E. siliculosus* was decoded and its carbohydrate metabolism was studied using phylogenetic approaches [65], [66]. Unlike green algae, *E. siliculosus* is not *Viridiplantae* (green plants) and it contains all NSE families except for UXE, further supporting their early divergence in the evolution. Since the cell wall components of *E. siliculosus* differ significantly than that of green plants, the NSE families in this organism must be involved in the synthesis of precursors for other carbohydrate polymers.

### Conclusion

This study represents the first systematic phylogenomic analysis of plant NSE families. We presented evidence that 1) different plant NSE families are distantly related and their progenitor genes diverged in ancient prokaryotic world before eukaryotes evolved; 2) plant UGlcAE genes may have a *Chlamydiae* bacterial progenitor; 3) the bi-domain RHM genes are only found in plants and *Nematoda*, and any fungi and unicellular eukaryotic organisms that encode a 3,5-epimerase-4-reductase gene also have a separate 4,6-dehydratase gene while some other eukaryotes only encode the 4,6-dehydratase genes; and 4) the bi-domain RHM genes evolved through a gene fusion event happened in early eukaryotes while NRS/ER genes may have evolved later from RHM genes by losing the N-terminal domain. Based on these findings, we proposed an evolutionary model for the origin and evolution of NSE families in nature.

## Materials and Methods

### Data sources

Predicted open reading frames of four plant and algal genomes were downloaded from various places: *Chlamydomonas reinhardtii* v3.1 [28] from ftp://ftp.jgi-psf.org/pub/JGI_data/Chlamy/v3.1/, *Physcomitrella patens ssp patens* v1.1 [29] from ftp://ftp.jgi-psf.org/pub/JGI_data/Physcomitrella_patens/v1.1/, *Oryza sativa* v6.1 [30], [31] from ftp://ftp.plantbiology.msu.edu/pub/data/Eukaryotic_Projects/o_sativa/annotation_dbs/pseudomolecules/version_6.1/ and *Arabidopsis thaliana* v9.0 [32] from ftp://ftp.arabidopsis.org/Sequences/blast_datasets/TAIR9_blastsets/. The NCBI-nr database was downloaded from ftp://ftp.ncbi.nih.gov/blast/db/FASTA/ as of Dec. 09, 2009. Most proteins of the four plant and algal genomes are included by NCBI-nr database. Protein IDs from the genome release file are mapped to GenBank IDs by doing blastp search. For proteins that are not in NCBI-nr, blastn search is performed to find the best UniGene ID or EST ID (Fig. 2).

### HMMER search

The *hmmsearch* command of the HMMER package [67] is used to search Pfam HMMs or self-built HMMs in ls mode (global with respect to query domain and local with respect to hit protein [67]) against protein databases. Unless otherwise indicated, an E-value cutoff <1e-2 is used to select significant protein homologs.

### HMM building

To generate an HMM model, homologous sequences are collected and a multiple sequence alignment (MSA) is created by using the MAFFT v6.717 program [68]. The MSA is further processed by the *hmmbuild* and the *hmmcalibrate* commands in the HMMER package to develop an HMM model, which could be used for later homology searches.

### Phylogenetic analyses

MSAs were performed using the MAFFT v6.717 program [68]. For Figures 2 and 3, PhyML v3.0 program [69] was used to perform phylogeny reconstruction with the following parameters: JTT model, 100 replicates of bootstrap analyses, estimated proportion of invariable sites, four rate categories, estimated gamma distribution parameter, and optimized starting BIONJ tree. For the other phylogenies, FastTree v2.1.1 program was used [70], which implements an ultra fast and accurate approximate maximum likelihood method. The accuracy of FastTree v2.1.1 phylogeny is considered to be slightly better than PhyML v3.0 [69] with NNI (minimum-evolution nearest-neighbor interchanges) moves, and is 100-1,000 times faster and requires much less computer memory [70]. FastTree analyses were conducted with default parameters; specifically, the amino acid substitution matrix is JTT, the number of rate categories of sites (CAT model) is 20, the local support values of each node are computed by resampling the site likelihoods 1,000 times and performing the Shimodaira Hasegawa test.

## Supporting Information

Figure S1 **Phylogeny of 257 plant** ***Epimerase*** **domains (the complete version of** **Figure 2** **).**
(PDF)

Figure S2 **Phylogeny of the close homologs of plant UGlcAE proteins.** The Epimerase domains of 157 proteins are used in generating a multiple sequence alignment. Based on that the phylogeny is built using FastTree v2.1.1 and displayed using the Interactive Tree of Life (iTOL) web server. Selected supporting values >70% are shown. Sequences are indicated using GenBank gi numbers followed by species names followed by taxonomy ranks. More information about these proteins could be found in Table S3.
(PDF)

Figure S3 **Phylogeny of 254 RHM N-terminals (the complete version of** **Figure 4A** **).** More information about these proteins could be found in Table S4.
(PDF)

Figure S4 **Phylogeny of 78 RHM C-terminals (the complete version of** **Figure 4B** **).** More information about these proteins could be found in Table S5.
(PDF)

Figure S5 **Phylogeny of the close homologs of plant UXS proteins.** The Epimerase domains of 311 proteins are used in generating a multiple sequence alignment. Based on that the phylogeny is built using FastTree v2.1.1 and displayed using the Interactive Tree of Life (iTOL) web server. Selected supporting values >70% are shown. Sequences are indicated using GenBank gi numbers followed by species names followed by taxonomy ranks. More information about these proteins could be found in Table S6.
(PDF)

Figure S6 **Phylogeny of the close homologs of plant UAXS (AXS) proteins.** The Epimerase domains of 55 proteins are used in generating a multiple sequence alignment. Based on that the phylogeny is built using FastTree v2.1.1 and displayed using the Interactive Tree of Life (iTOL) web server. Selected supporting values >70% are shown. Sequences are indicated using GenBank gi numbers followed by species names followed by taxonomy ranks. More information about these proteins could be found in Table S7.
(PDF)

Figure S7 **Phylogeny of the close homologs of plant MAR proteins.** The Epimerase domains of 52 proteins are used in generating a multiple sequence alignment. Based on that the phylogeny is built using FastTree v2.1.1 and displayed using the Interactive Tree of Life (iTOL) web server. Selected supporting values >70% are shown. Sequences are indicated using GenBank gi numbers followed by species names followed by taxonomy ranks. More information about these proteins could be found in Table S8.
(PDF)

Figure S8 **Phylogeny of the close homologs of plant GME proteins.** The Epimerase domains of 121 proteins are used in generating a multiple sequence alignment. Based on that the phylogeny is built using FastTree v2.1.1 and displayed using the Interactive Tree of Life (iTOL) web server. Selected supporting values >70% are shown. Sequences are indicated using GenBank gi numbers followed by species names followed by taxonomy ranks. More information about these proteins could be found in Table S9.
(PDF)

Figure S9 **Phylogeny of the close homologs of plant GMD proteins.** The Epimerase domains of 69 proteins are used in generating a multiple sequence alignment. Based on that the phylogeny is built using FastTree v2.1.1 and displayed using the Interactive Tree of Life (iTOL) web server. Selected supporting values >70% are shown. Sequences are indicated using GenBank gi numbers followed by species names followed by taxonomy ranks. More information about these proteins could be found in Table S10.
(PDF)

Figure S10 **Phylogeny of the close homologs of plant SQD1 proteins.** The Epimerase domains of 92 proteins are used in generating a multiple sequence alignment. Based on that the phylogeny is built using FastTree v2.1.1 and displayed using the Interactive Tree of Life (iTOL) web server. Selected supporting values >70% are shown. Sequences are indicated using GenBank gi numbers followed by species names followed by taxonomy ranks. More information about these proteins could be found in Table S11.
(PDF)

Figure S11 **Phylogeny of the close homologs of plant GER proteins.** The Epimerase domains of 61 proteins are used in generating a multiple sequence alignment. Based on that the phylogeny is built using FastTree v2.1.1 and displayed using the Interactive Tree of Life (iTOL) web server. Selected supporting values >70% are shown. Sequences are indicated using GenBank gi numbers followed by species names followed by taxonomy ranks. More information about these proteins could be found in Table S12.
(PDF)

Figure S12 **Phylogeny of the close homologs of plant UXE proteins.** The Epimerase domains of 78 proteins are used in generating a multiple sequence alignment. Based on that the phylogeny is built using FastTree v2.1.1 and displayed using the Interactive Tree of Life (iTOL) web server. Selected supporting values >70% are shown. Sequences are indicated using GenBank gi numbers followed by species names followed by taxonomy ranks. More information about these proteins could be found in Table S13.
(PDF)

Figure S13 **Phylogeny of the close homologs of plant UGE proteins.** The Epimerase domains of 220 proteins are used in generating a multiple sequence alignment. Based on that the phylogeny is built using FastTree v2.1.1 and displayed using the Interactive Tree of Life (iTOL) web server. Selected supporting values >70% are shown. Sequences are indicated using GenBank gi numbers followed by species names followed by taxonomy ranks. More information about these proteins could be found in Table S14.
(PDF)

Figure S14 **Phylogeny of the close homologs of plant CRB proteins.** The Epimerase domains of 65 proteins are used in generating a multiple sequence alignment. Based on that the phylogeny is built using FastTree v2.1.1 and displayed using the Interactive Tree of Life (iTOL) web server. Selected supporting values >70% are shown. Sequences are indicated using GenBank gi numbers followed by species names followed by taxonomy ranks. More information about these proteins could be found in Table S15.
(PDF)

Figure S15 **Phylogeny of the close homologs of plant UGD proteins.** The Arabidopsis UGD proteins were used to search against sequenced plants to identify close homologs, which were collected and aligned to build an HMM. The HMM was further used to search against the NCIB-nr database. All proteins homologs with E-value <1e-2 were collected and aligned. Based on the alignment the phylogeny is built using FastTree v2.1.1, and the sub-tree containing 273 sequences closest to plant UGD proteins is displayed using the Interactive Tree of Life (iTOL) web server. Selected supporting values >70% are shown. Sequences are indicated using GenBank gi numbers followed by the protein region that is aligned to the plant UGD HMM, followed by species names and taxonomy ranks. More information about these proteins could be found in Table S16.
(PDF)

Table S1 **Phyletic information of the closest bacterial homologs of plant NDP-sugar biosynthetic enzymes.**
(XLS)

Table S2 **Prokaryotic proteins homologous to GenBank protein gi#:268680838 (from Sulfurospirillum deleyianum DSM 6946, shown as a red star in** **Fig. 4A** **).**
(XLS)

Table S3 **NCBI-nr proteins most homologous to plant UGlcAE HMM.**
(XLS)

Table S4 **NCBI-nr proteins most homologous to plant RHM-N HMM.**
(XLS)

Table S5 **NCBI-nr proteins homologous to a self-built plant NRS/ER HMM.**
(XLS)

Table S6 **NCBI-nr proteins most homologous to plant UXS HMM.**
(XLS)

Table S7 **NCBI-nr proteins most homologous to plant UAXS HMM.**
(XLS)

Table S8 **NCBI-nr proteins most homologous to plant MAR HMM.**
(XLS)

Table S9 **NCBI-nr proteins most homologous to plant GME HMM.**
(XLS)

Table S10 **NCBI-nr proteins most homologous to plant GMD HMM.**
(XLS)

Table S11 **NCBI-nr proteins most homologous to plant SQD1 HMM.**
(XLS)

Table S12 **NCBI-nr proteins most homologous to plant GER HMM.**
(XLS)

Table S13 **NCBI-nr proteins most homologous to plant UXE HMM.**
(XLS)

Table S14 **NCBI-nr proteins most homologous to plant UGE HMM.**
(XLS)

Table S15 **NCBI-nr proteins most homologous to plant CRB HMM.**
(XLS)

Table S16 **NCBI-nr proteins homologous to plant UGD HMM.**
(XLS)
