# Supplementary figures and images for: Evolution of Plant Nucleotide-Sugar Interconversion Enzymes
Source: PLoS One. 2011 Nov 18;6(11):e27995. doi: 10.1371/journal.pone.0027995 (PMC3220709; doi:10.1371/journal.pone.0027995)

Fig. S2: UGlcAE

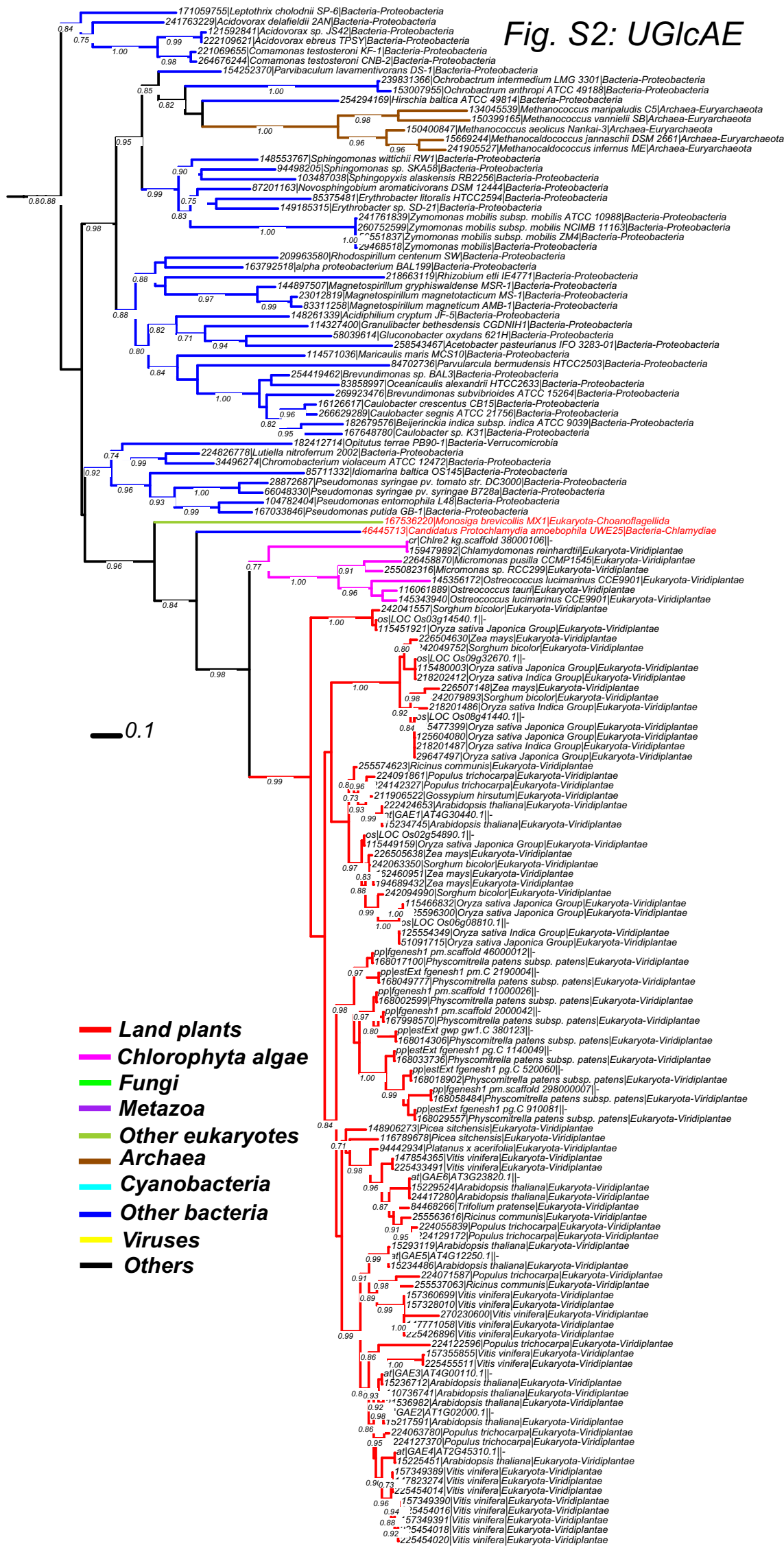

Supplement: Figure S2 — Phylogeny of the close homologs of plant UGlcAE proteins. The Epimerase domains of 157 proteins are used in generating a multiple sequence alignment. Based on that the phylogeny is built using FastTree v2.1.1 and displayed using the Interactive Tree of Life (iTOL) web server. Selected supporting values >70% are shown. Sequences are indicated using GenBank gi numbers followed by species names followed by taxonomy ranks. More information about these proteins could be found in Table S3. (PDF) [file pone.0027995.s002.pdf]

# Fig. S3: RHM-N

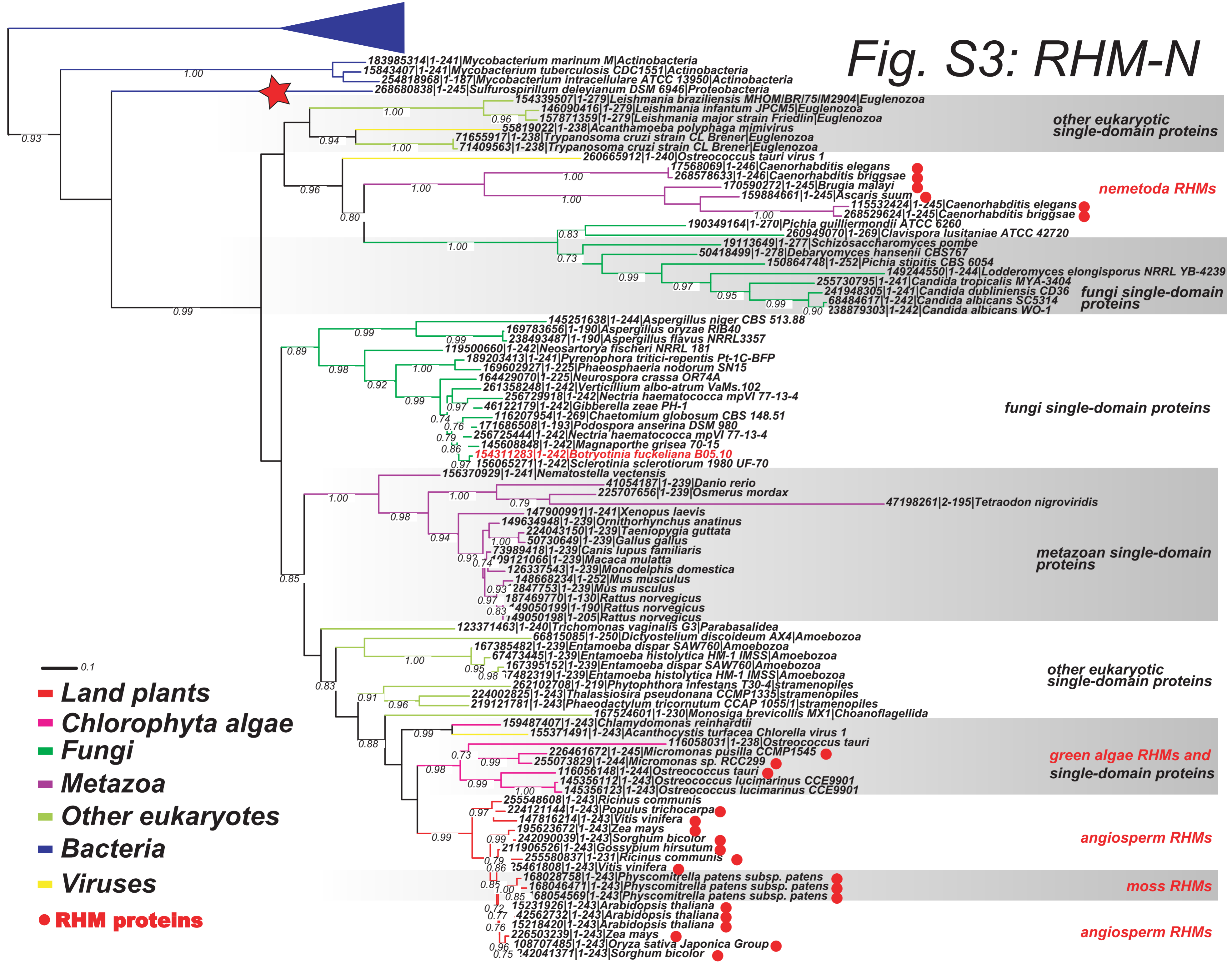

Supplement: Figure S3 — Phylogeny of 254 RHM N-terminals (the complete version of Figure 4A ). More information about these proteins could be found in Table S4. (PDF) [file pone.0027995.s003.pdf]

Fig. S4: RHM-C

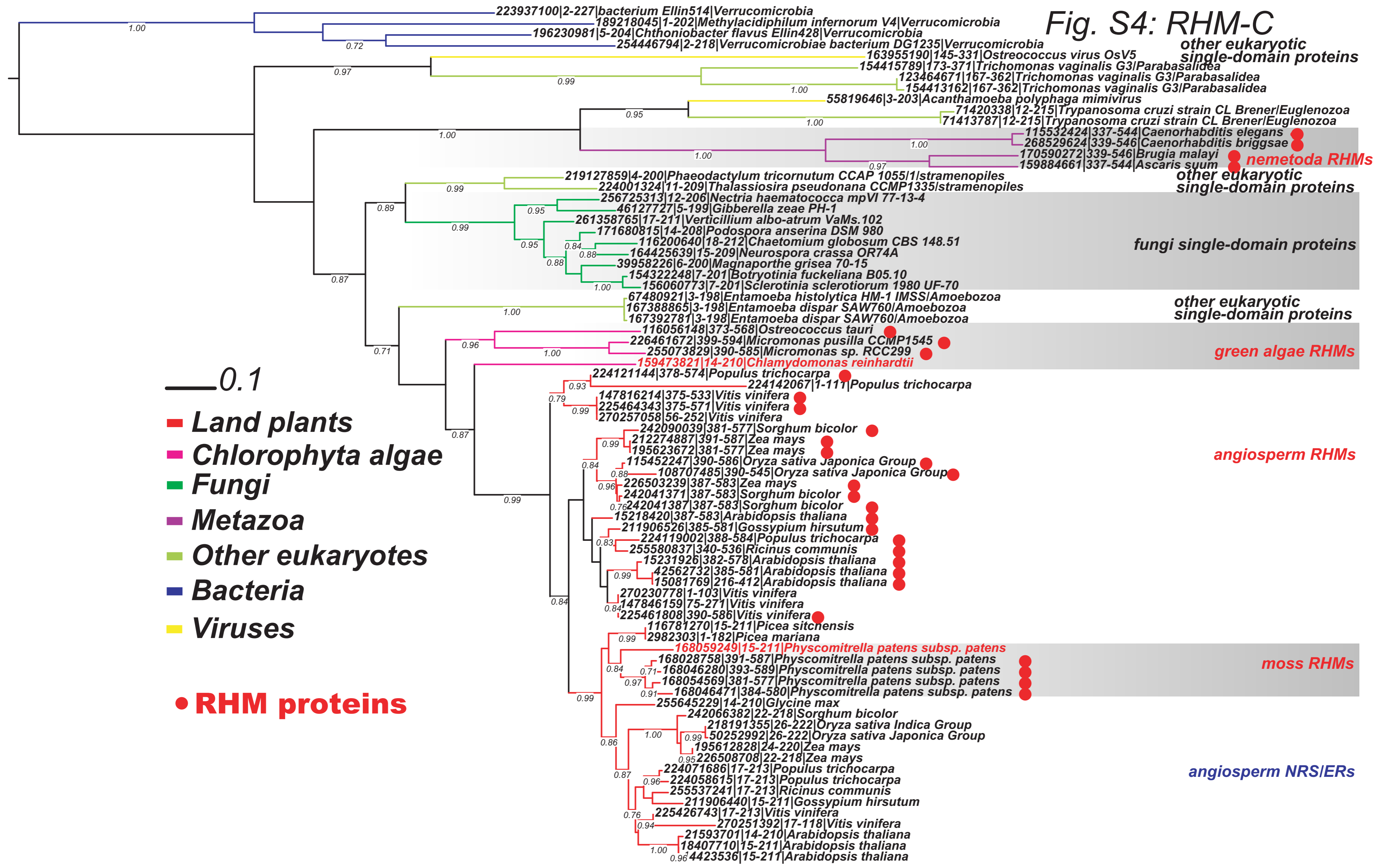

Supplement: Figure S4 — Phylogeny of 78 RHM C-terminals (the complete version of Figure 4B ). More information about these proteins could be found in Table S5. (PDF) [file pone.0027995.s004.pdf]

Fig. S5: UXS

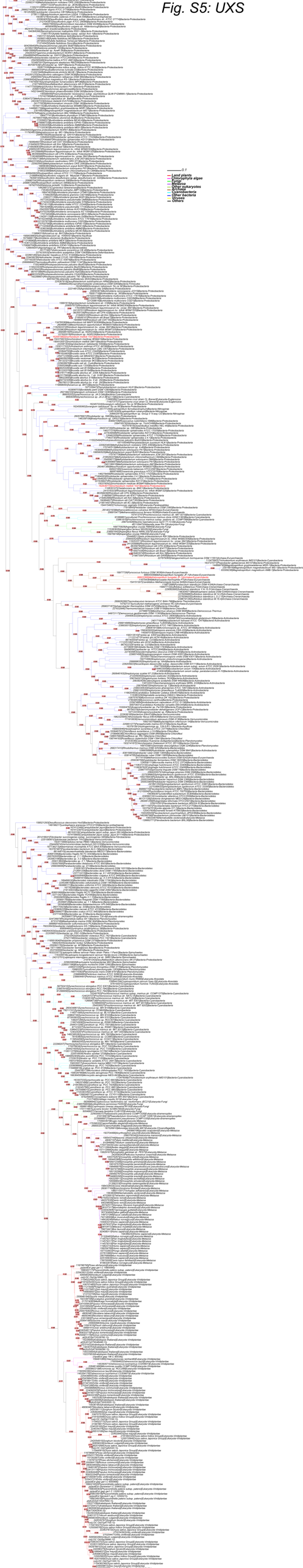

Supplement: Figure S5 — Phylogeny of the close homologs of plant UXS proteins. The Epimerase domains of 311 proteins are used in generating a multiple sequence alignment. Based on that the phylogeny is built using FastTree v2.1.1 and displayed using the Interactive Tree of Life (iTOL) web server. Selected supporting values >70% are shown. Sequences are indicated using GenBank gi numbers followed by species names followed by taxonomy ranks. More information about these proteins could be found in Table S6. (PDF) [file pone.0027995.s005.pdf]

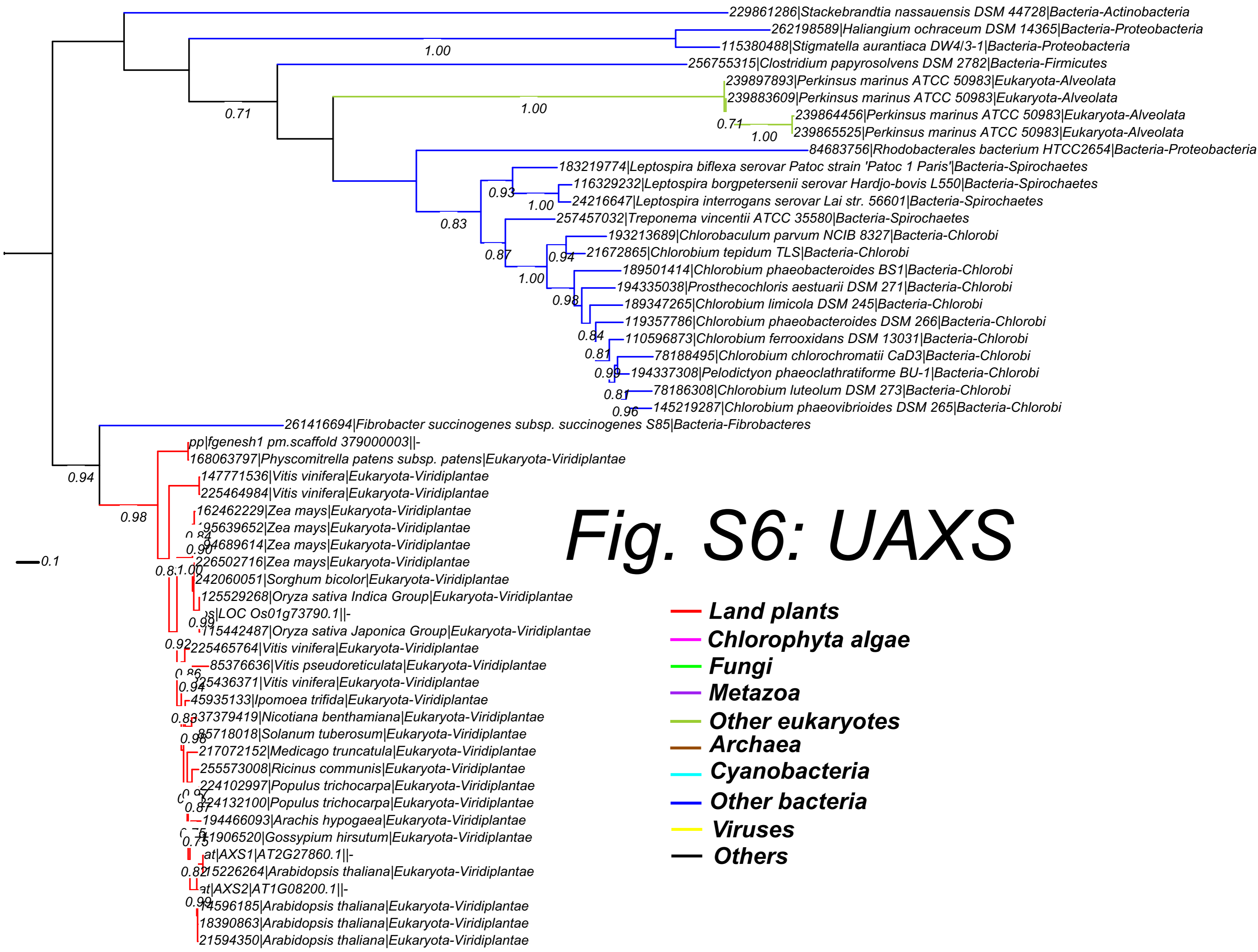

Supplement: Figure S6 — Phylogeny of the close homologs of plant UAXS (AXS) proteins. The Epimerase domains of 55 proteins are used in generating a multiple sequence alignment. Based on that the phylogeny is built using FastTree v2.1.1 and displayed using the Interactive Tree of Life (iTOL) web server. Selected supporting values >70% are shown. Sequences are indicated using GenBank gi numbers followed by species names followed by taxonomy ranks. More information about these proteins could be found in Table S7. (PDF) [file pone.0027995.s006.pdf]

# Fig. S7: MAR

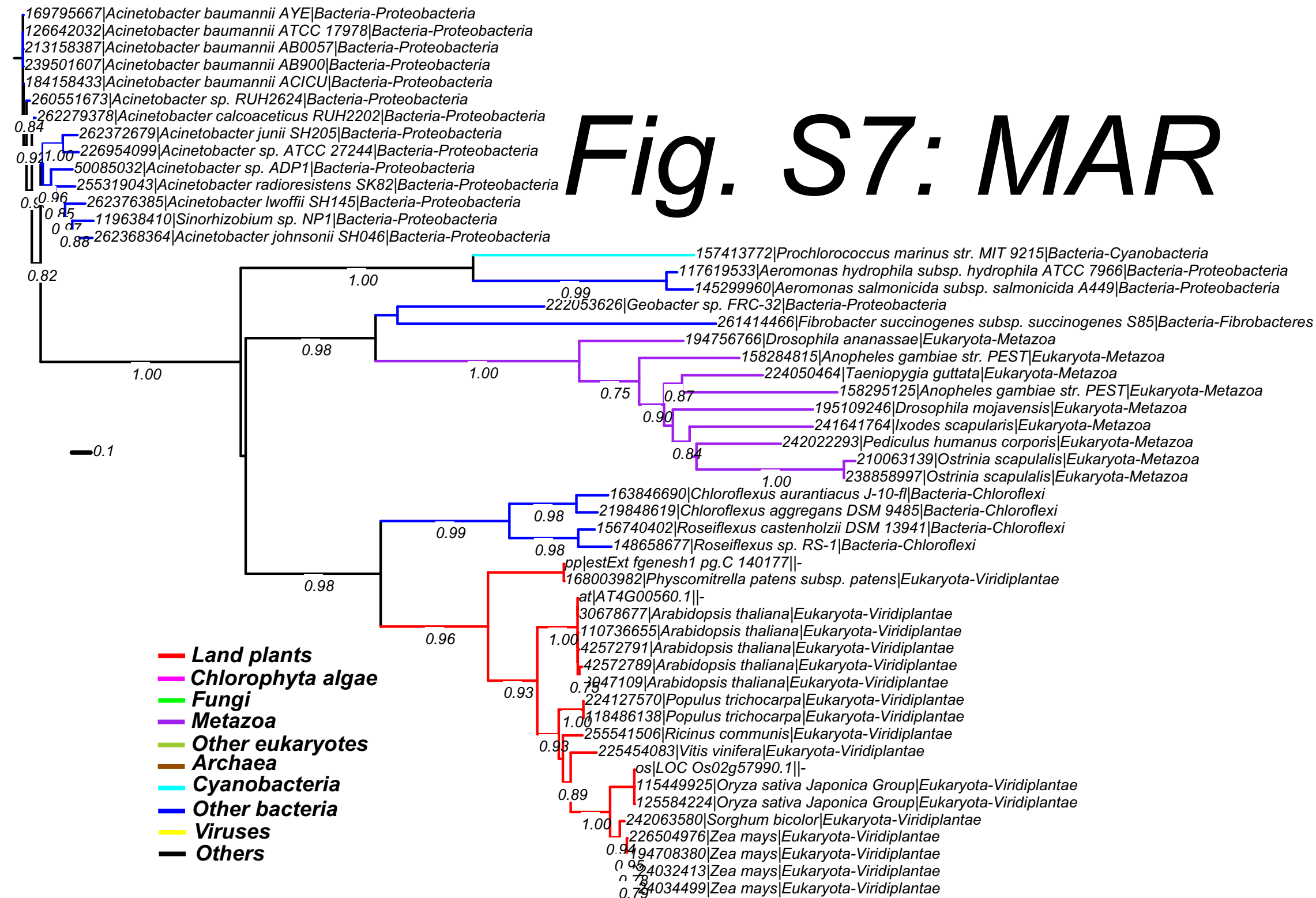

Supplement: Figure S7 — Phylogeny of the close homologs of plant MAR proteins. The Epimerase domains of 52 proteins are used in generating a multiple sequence alignment. Based on that the phylogeny is built using FastTree v2.1.1 and displayed using the Interactive Tree of Life (iTOL) web server. Selected supporting values >70% are shown. Sequences are indicated using GenBank gi numbers followed by species names followed by taxonomy ranks. More information about these proteins could be found in Table S8. (PDF) [file pone.0027995.s007.pdf]

Fig. S8: GME

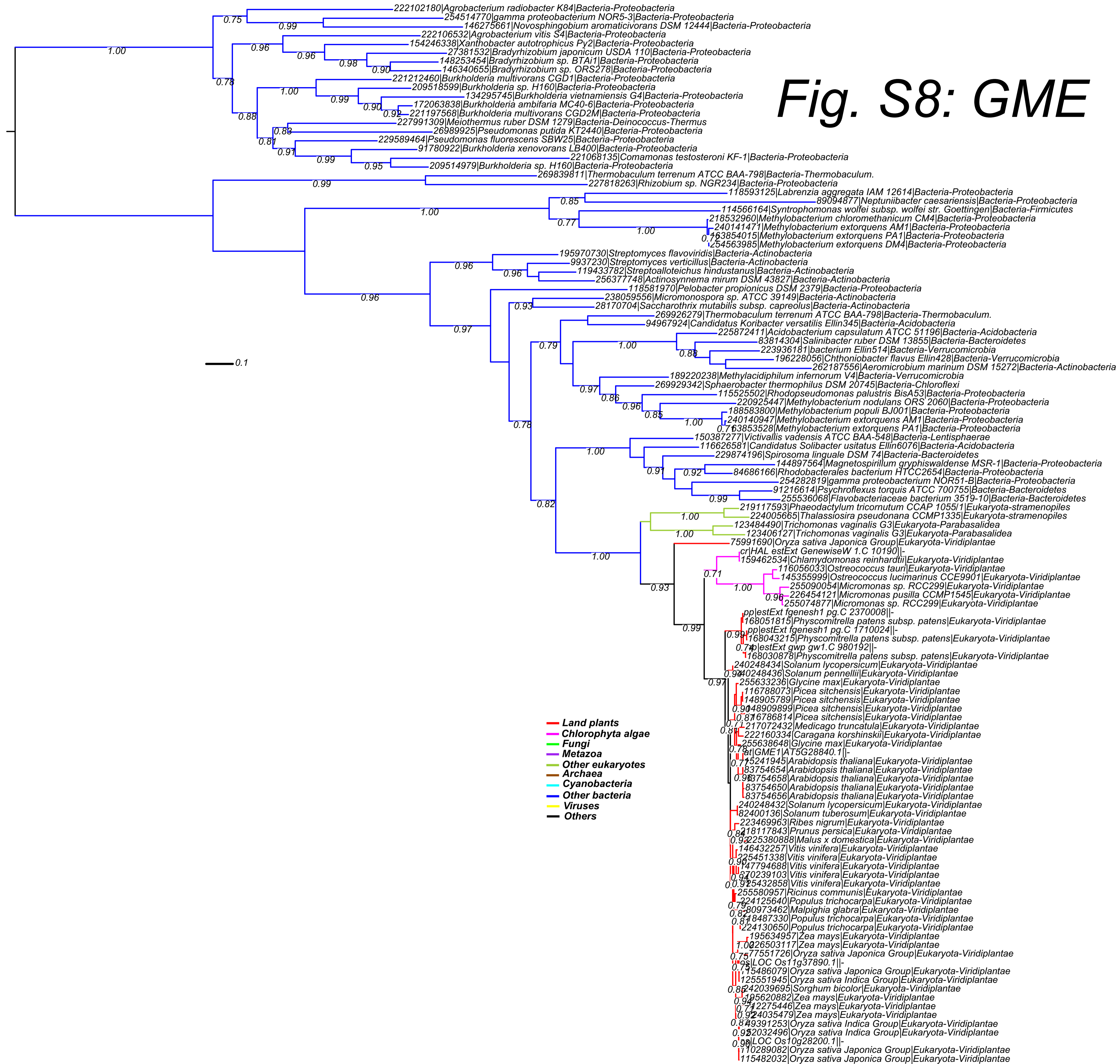

Supplement: Figure S8 — Phylogeny of the close homologs of plant GME proteins. The Epimerase domains of 121 proteins are used in generating a multiple sequence alignment. Based on that the phylogeny is built using FastTree v2.1.1 and displayed using the Interactive Tree of Life (iTOL) web server. Selected supporting values >70% are shown. Sequences are indicated using GenBank gi numbers followed by species names followed by taxonomy ranks. More information about these proteins could be found in Table S9. (PDF) [file pone.0027995.s008.pdf]

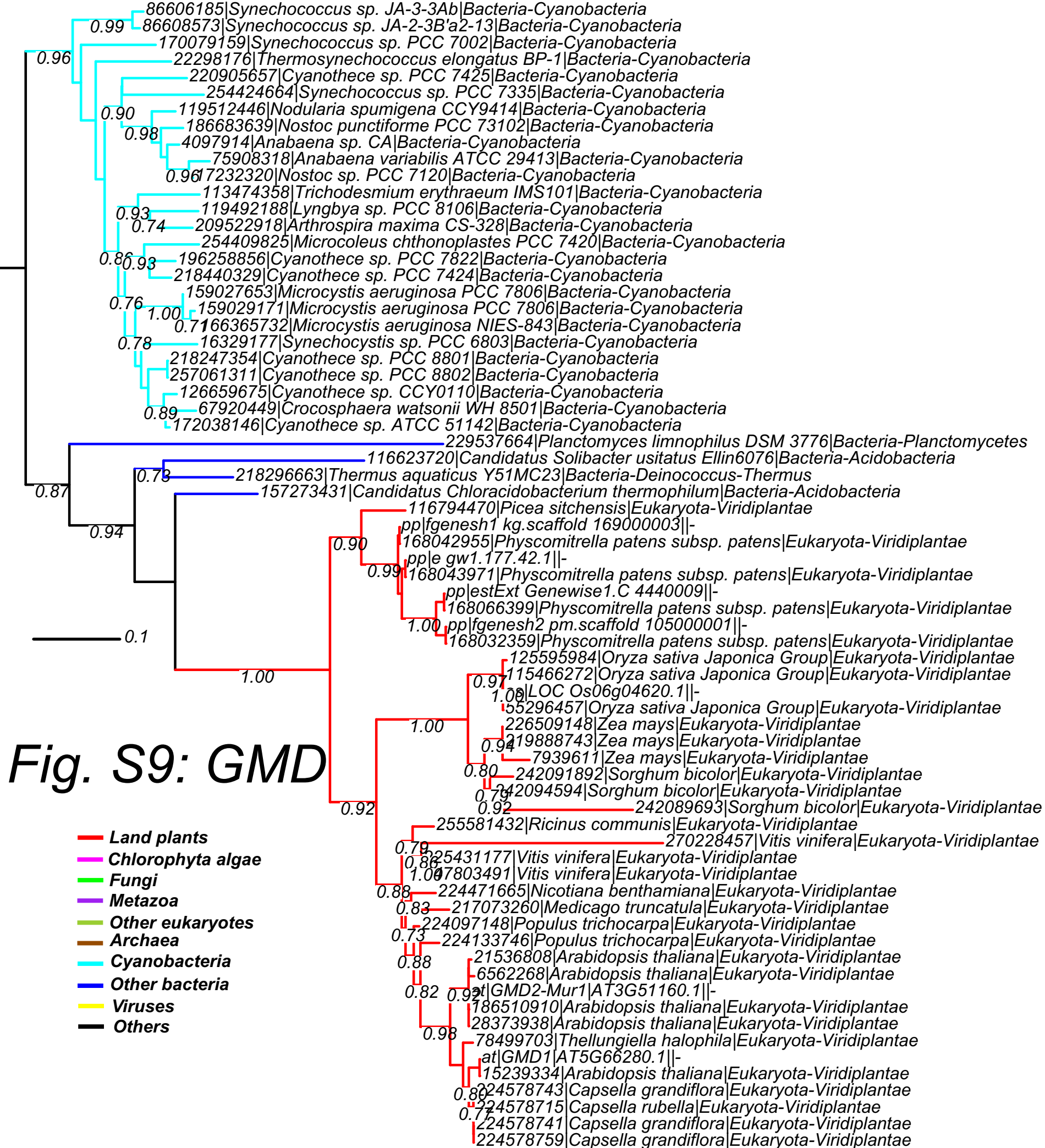

Supplement: Figure S9 — Phylogeny of the close homologs of plant GMD proteins. The Epimerase domains of 69 proteins are used in generating a multiple sequence alignment. Based on that the phylogeny is built using FastTree v2.1.1 and displayed using the Interactive Tree of Life (iTOL) web server. Selected supporting values >70% are shown. Sequences are indicated using GenBank gi numbers followed by species names followed by taxonomy ranks. More information about these proteins could be found in Table S10. (PDF) [file pone.0027995.s009.pdf]

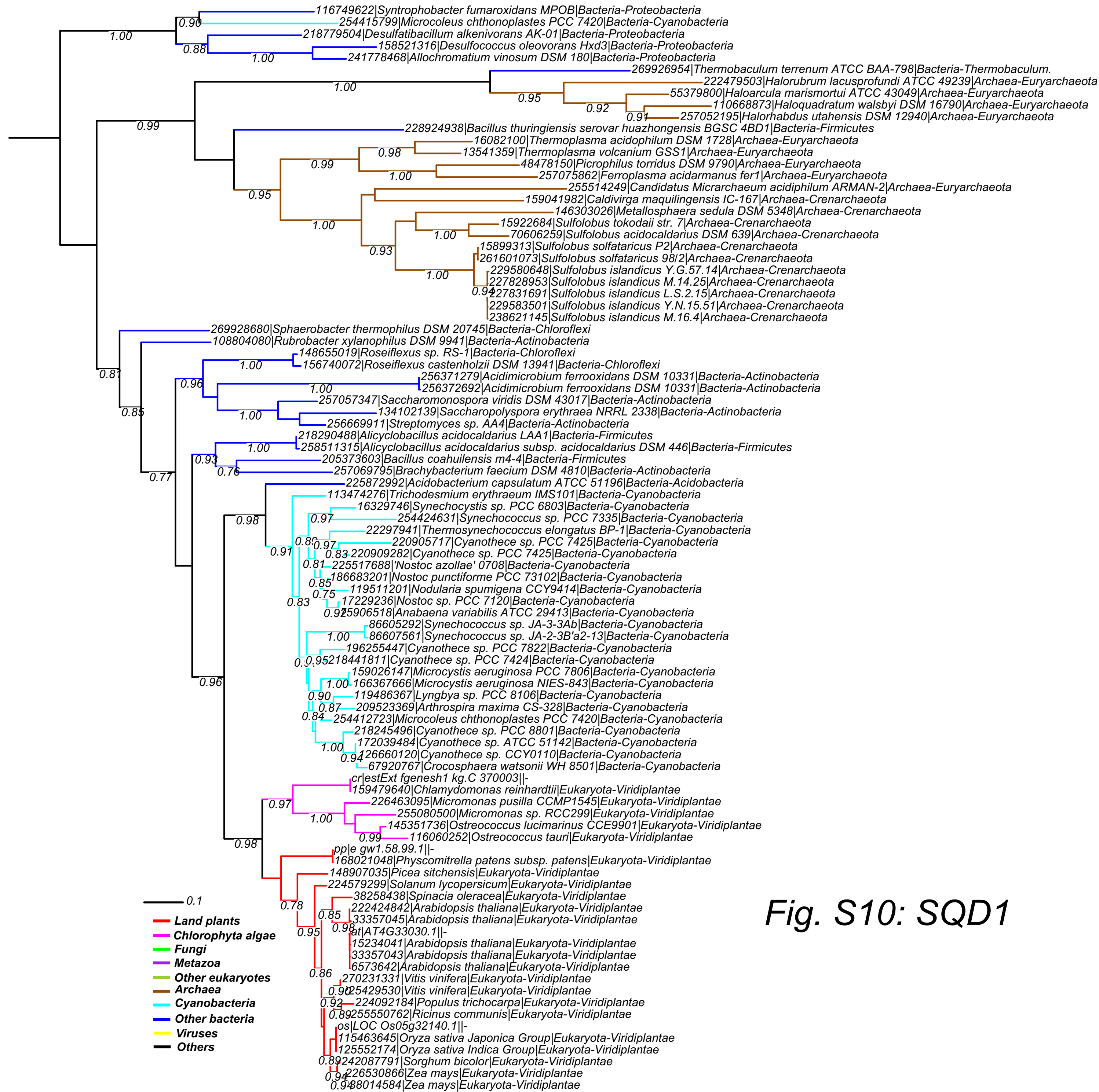

Fig. S10: SQD1

Supplement: Figure S10 — Phylogeny of the close homologs of plant SQD1 proteins. The Epimerase domains of 92 proteins are used in generating a multiple sequence alignment. Based on that the phylogeny is built using FastTree v2.1.1 and displayed using the Interactive Tree of Life (iTOL) web server. Selected supporting values >70% are shown. Sequences are indicated using GenBank gi numbers followed by species names followed by taxonomy ranks. More information about these proteins could be found in Table S11. (PDF) [file pone.0027995.s010.pdf]

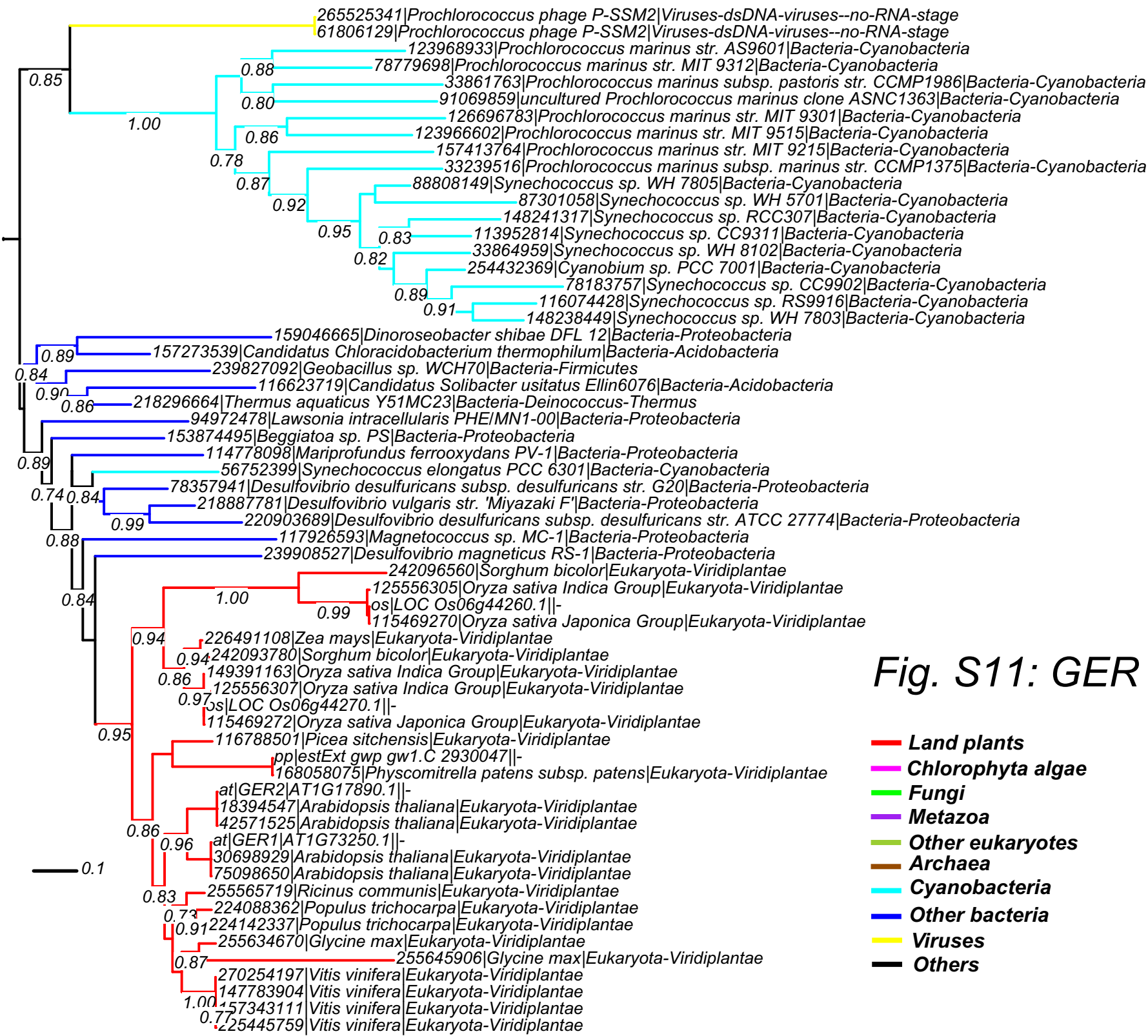

Fig. S11: GER

Supplement: Figure S11 — Phylogeny of the close homologs of plant GER proteins. The Epimerase domains of 61 proteins are used in generating a multiple sequence alignment. Based on that the phylogeny is built using FastTree v2.1.1 and displayed using the Interactive Tree of Life (iTOL) web server. Selected supporting values >70% are shown. Sequences are indicated using GenBank gi numbers followed by species names followed by taxonomy ranks. More information about these proteins could be found in Table S12. (PDF) [file pone.0027995.s011.pdf]

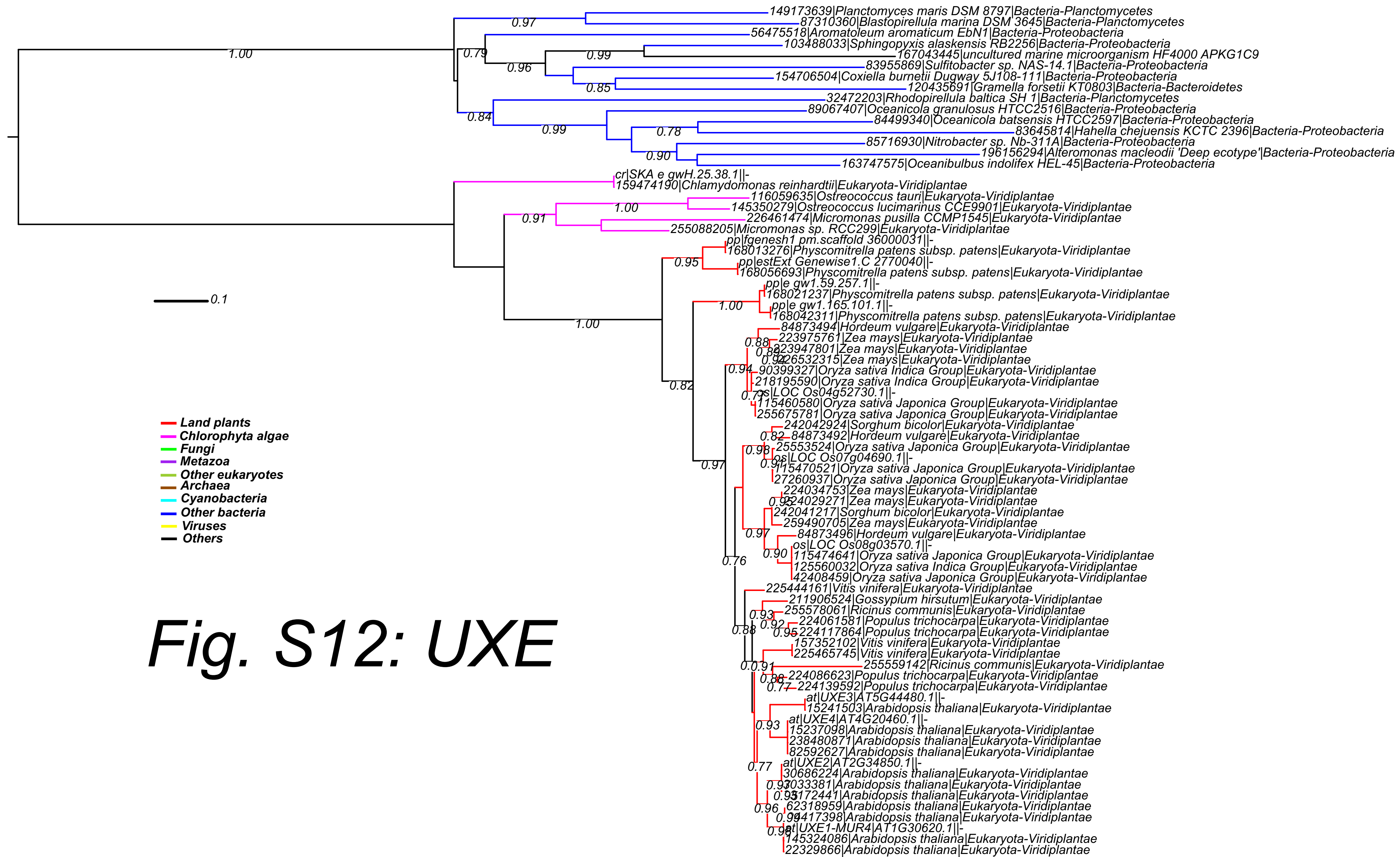

Supplement: Figure S12 — Phylogeny of the close homologs of plant UXE proteins. The Epimerase domains of 78 proteins are used in generating a multiple sequence alignment. Based on that the phylogeny is built using FastTree v2.1.1 and displayed using the Interactive Tree of Life (iTOL) web server. Selected supporting values >70% are shown. Sequences are indicated using GenBank gi numbers followed by species names followed by taxonomy ranks. More information about these proteins could be found in Table S13. (PDF) [file pone.0027995.s012.pdf]

Fig. S13: UGE

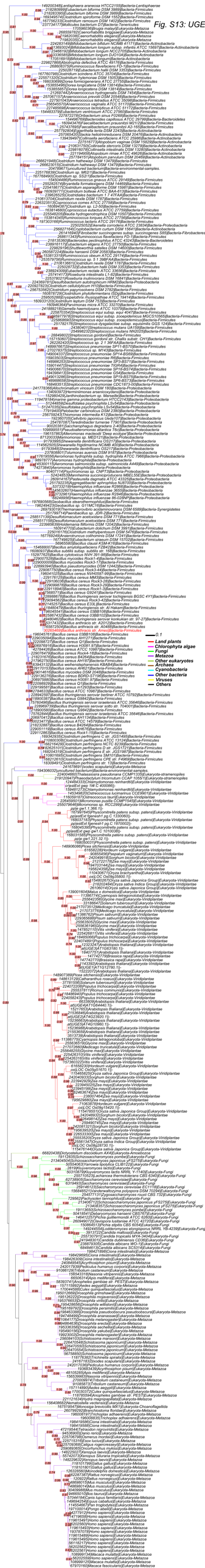

Supplement: Figure S13 — Phylogeny of the close homologs of plant UGE proteins. The Epimerase domains of 220 proteins are used in generating a multiple sequence alignment. Based on that the phylogeny is built using FastTree v2.1.1 and displayed using the Interactive Tree of Life (iTOL) web server. Selected supporting values >70% are shown. Sequences are indicated using GenBank gi numbers followed by species names followed by taxonomy ranks. More information about these proteins could be found in Table S14. (PDF) [file pone.0027995.s013.pdf]

Fig. S14: CRB

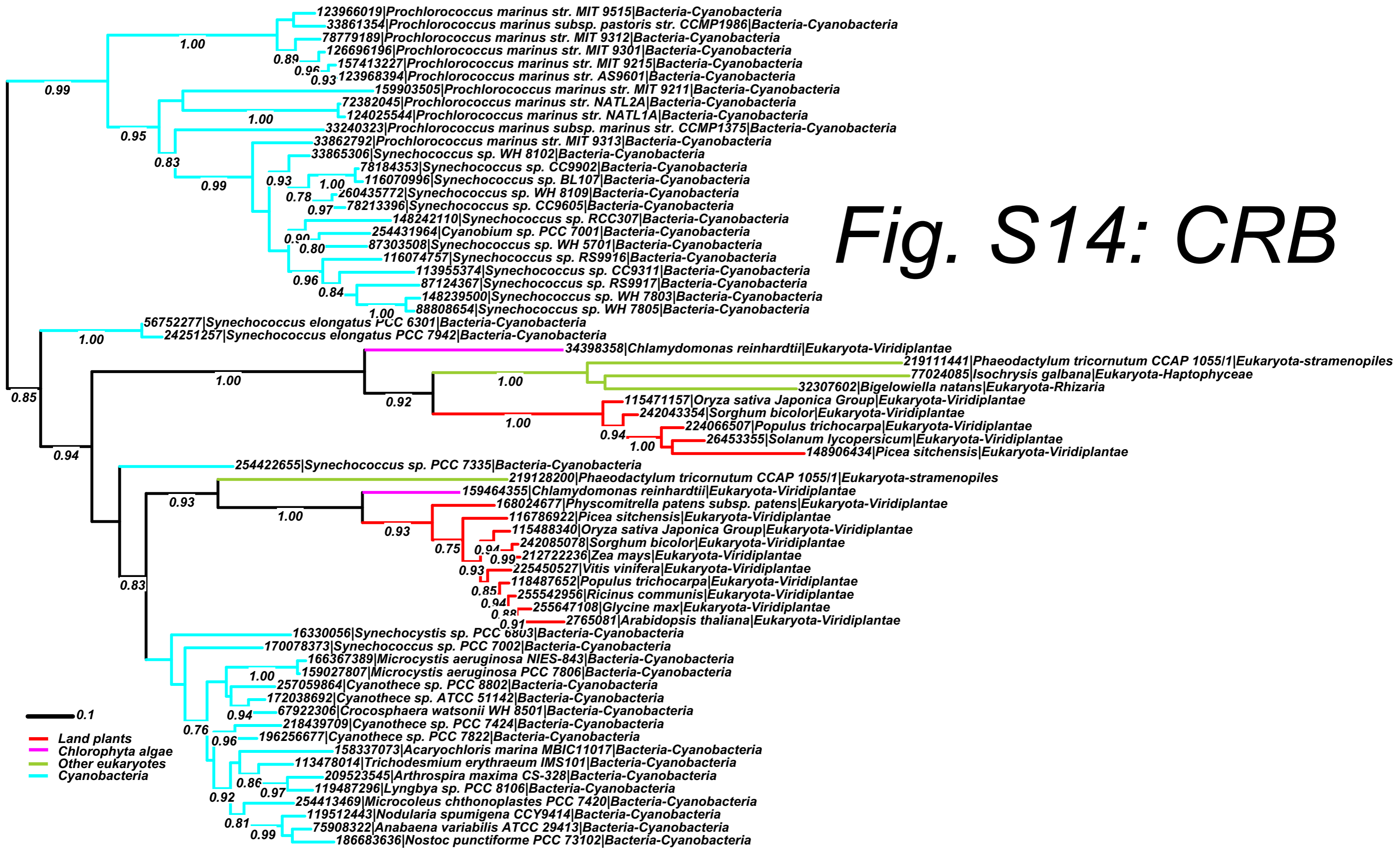

Supplement: Figure S14 — Phylogeny of the close homologs of plant CRB proteins. The Epimerase domains of 65 proteins are used in generating a multiple sequence alignment. Based on that the phylogeny is built using FastTree v2.1.1 and displayed using the Interactive Tree of Life (iTOL) web server. Selected supporting values >70% are shown. Sequences are indicated using GenBank gi numbers followed by species names followed by taxonomy ranks. More information about these proteins could be found in Table S15. (PDF) [file pone.0027995.s014.pdf]

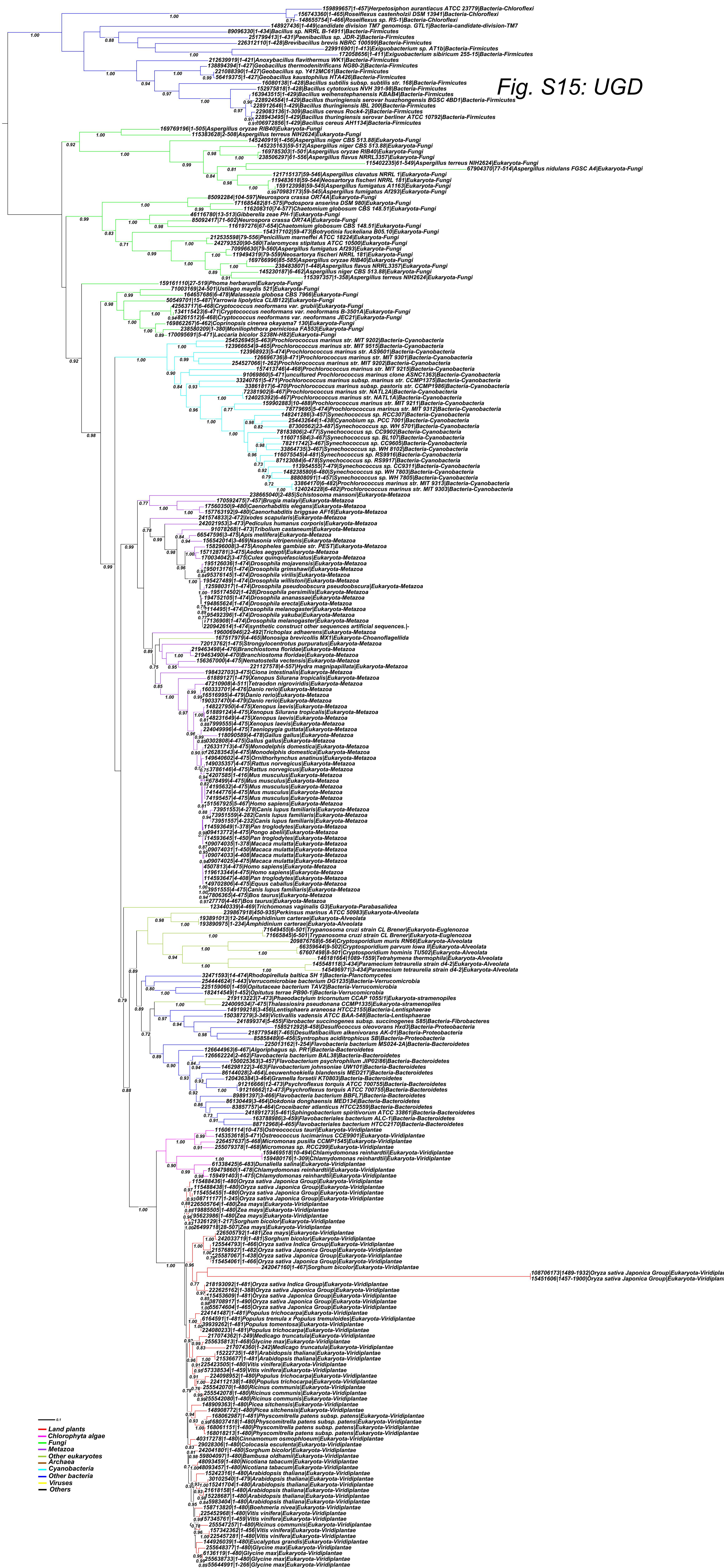

Supplement: Figure S15 — Phylogeny of the close homologs of plant UGD proteins. The Arabidopsis UGD proteins were used to search against sequenced plants to identify close homologs, which were collected and aligned to build an HMM. The HMM was further used to search against the NCIB-nr database. All proteins homologs with E-value <1e-2 were collected and aligned. Based on the alignment the phylogeny is built using FastTree v2.1.1, and the sub-tree containing 273 sequences closest to plant UGD proteins is displayed using the Interactive Tree of Life (iTOL) web server. Selected supporting values >70% are shown. Sequences are indicated using GenBank gi numbers followed by the protein region that is aligned to the plant UGD HMM, followed by species names and taxonomy ranks. More information about these proteins could be found in Table S16. (PDF) [file pone.0027995.s015.pdf]
