# Supplementary material for: Evolution of Plant Nucleotide-Sugar Interconversion Enzymes
Source: PLoS One. 2011 Nov 18;6(11):e27995. doi: 10.1371/journal.pone.0027995 (PMC3220709; doi:10.1371/journal.pone.0027995)
Supplement: Figure S1 — Phylogeny of 257 plant Epimerase domains (the complete version of Figure 2 ). (PDF) [file pone.0027995.s001.pdf]

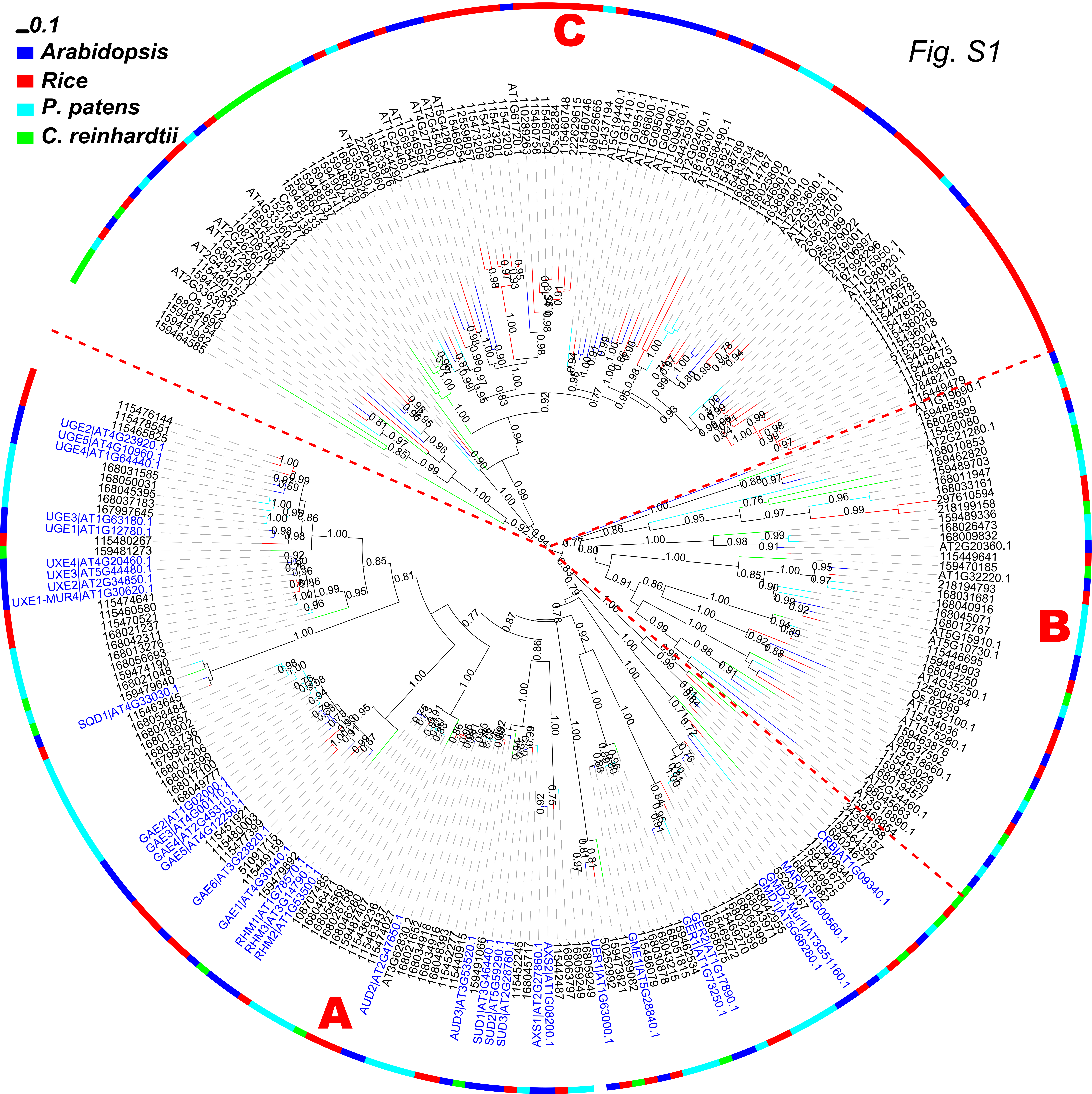

0.1
Arabidopsis
Rice
P. patens
C. reinhardtii
Fig. S1
C
B
A
UGE2|AT4G23920.1
UGE5|AT4G10960.1
UGE4|AT1G64440.1
UGE3|AT1G63180.1
UGE1|AT1G12780.1
UXE4|AT4G20460.1
UXE3|AT5G44480.1
UXE2|AT2G34850.1
UXE1-MUR4|AT1G30620.1
SQD1|AT4G33030.1
GAE2|AT1G02000.1
GAE3|AT4G00110.1
GAE4|AT2G45310.1
GAE5|AT4G12250.1
GAE6|AT3G23820.1
GAE1|AT4G30440.1
RHM1|AT1G78570.1
RHM3|AT3G14790.1
RHM2|AT1G53500.1
AUD2|AT2G47650.1
AUD3|AT3G53520.1
SUD1|AT3G46440.1
SUD2|AT5G59290.1
SUD3|AT2G28760.1
AXS1|AT2G27860.1
AXS2|AT1G08200.1
UER1|AT1G63000.1
GME1|AT5G28840.1
GER2|AT1G17890.1
GER1|AT1G73250.1
GMD1|AT5G66280.1
GMD2-Mur1|AT3G51160.1
MAR|AT4G00560.1
CRB|AT1G09340.1
